# Beyond the Tumor: A Rare Paraneoplastic Combination Unmasking Lung Cancer

**DOI:** 10.7759/cureus.117588

**Published:** 2026-10-07

**Authors:** Mariame Jaouhari, Hanane Ikrou, Oussama Halloumi, Hind Serhane

**Affiliations:** 1 Department of Pulmonology, Mohamed VI University Hospital of Agadir, Agadir, MAR; 2 LARISS Laboratory, Faculty of Medicine and Pharmacy of Agadir, Ibn Zohr University, Agadir, MAR

**Keywords:** endocrine, lung cancer, paraneoplastic, prolonged aptt, syndromes

## Abstract

Paraneoplastic syndromes are heterogeneous clinical manifestations that may occasionally reveal an underlying malignancy. We report the case of a 37-year-old man with lung adenocarcinoma who presented with severe hypotonic hyponatremia suggestive of syndrome of inappropriate antidiuretic hormone secretion (SIADH), recurrent hypoglycemia, and an isolated prolonged activated partial thromboplastin time (aPTT), in whom a paraneoplastic origin of these abnormalities was suspected. This uncommon coexistence highlights the importance of considering an occult malignancy in patients presenting with multiple unexplained endocrine and coagulation abnormalities. It also illustrates the challenges of interpreting an isolated prolonged aPTT and the need for a systematic diagnostic evaluation of each abnormality before attributing it to a paraneoplastic mechanism.

## Introduction

Lung cancer is the leading cause of cancer-related mortality worldwide and has a major impact on patients' quality of life [1,2]. In 2022 alone, lung cancer accounted for an estimated 2.5 million new cases and 1.8 million deaths globally, making it both the most frequently diagnosed cancer and the leading cause of cancer death worldwide [2]. Although early screening strategies have improved, lung cancer is still frequently diagnosed at an advanced stage; diagnostic delay remains common and is associated with a higher stage at diagnosis and increased healthcare costs, making delayed diagnosis a persistent public health challenge [3].

Paraneoplastic syndromes (PNS) are clinical manifestations associated with malignancy that result from ectopic production of hormones or biologically active peptides by the tumor, or from immune cross-reactivity between malignant and normal tissue, independently of direct tumor invasion or metastatic spread [4,5]. They may precede the diagnosis of the underlying malignancy, and their timely recognition can lead to detection of an otherwise occult tumor at an earlier, more treatable stage [4]. For endocrine PNS in lung cancer, diagnostic criteria have been proposed, including an endocrine abnormality without physiological feedback regulation, absence of metastasis to the corresponding endocrine gland, and improvement after cancer treatment [5].

Syndrome of inappropriate antidiuretic hormone secretion (SIADH) is one of the most common endocrine PNS in lung cancer, but it is classically associated with small cell lung cancer and is uncommon in adenocarcinoma [5,6]. Non-islet-cell tumor hypoglycemia is rare and has mostly been described with large mesenchymal, adrenocortical or hepatic tumors [7,8]. An isolated prolonged activated partial thromboplastin time (aPTT) may result from factor deficiencies or acquired inhibitors, some of which have been reported in association with malignancy, and its misinterpretation may lead to unnecessary investigations or delayed invasive procedures [9,10].

The coexistence of SIADH-like hyponatremia, hypoglycemia and an unexplained coagulation abnormality in a patient with lung adenocarcinoma is therefore an unusual constellation. It poses a diagnostic challenge, because each abnormality may also have non-neoplastic causes, particularly in a severely ill patient. We report such a case, in which a paraneoplastic origin was suspected, and discuss the diagnostic reasoning required to evaluate each abnormality and the limits of this attribution.

## Case presentation

We report the case of a 37-year-old man with a history of smoking and current cannabis use, with no history of tuberculosis or recent exposure. He presented with a three-month history of progressive dyspnea (stage II), dry cough and abdominal pain. His condition worsened three days before admission with diarrhea, persistent vomiting, fever and a decline in general condition.

On clinical examination, the patient was obtunded, with a Glasgow score of 13/15 and a poor performance status (ECOG 4) [11]. He was cachectic, with a BMI of 13.6. He had a heart rate of 93 bpm, a blood pressure of 73/54 mmHg, a respiratory rate of 24 breaths/min and an oxygen saturation of 98% on room air; pulmonary auscultation was normal.

Initial investigations revealed severe hyponatremia (95 mmol/L), hypoglycemia (0.3 g/L), and hyperkalemia (6.81 mmol/L). Electrocardiography showed no significant electrical abnormalities (Figure 1).

**Figure 1:**
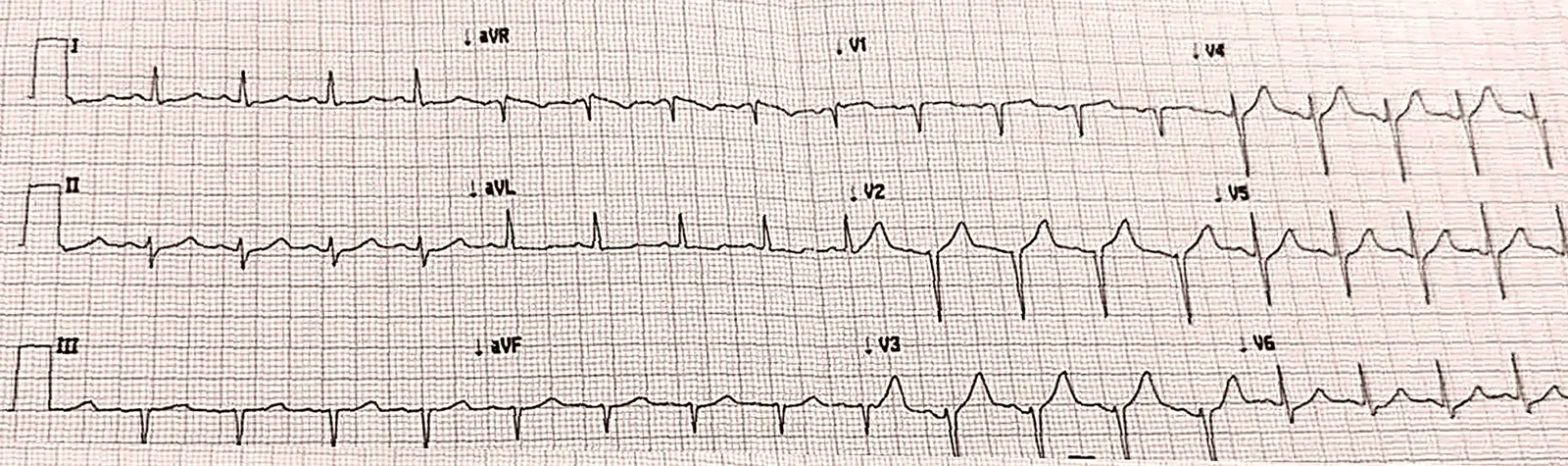
12-lead ECG showing sinus rhythm at 93 bpm, without peaked T waves, QRS widening or other electrical signs of hyperkalemia, despite a serum potassium of 6.81 mmol/L.

Further investigations revealed hypotonic hyponatremia, with a plasma osmolality of 201.61 mOsm/kg, a 24-hour urinary sodium of 150 mmol/24h, and a markedly low plasma uric acid (0.5 mg/dL). Serum cortisol (358 nmol/L) and thyroid-stimulating hormone (TSH) were within reference ranges. SIADH was suspected, supported by the low plasma uric acid level; however, urine osmolality and spot urine sodium were not available, and given the patient's hypotension, vomiting and diarrhea, a hypovolemic contribution could not be excluded. Adrenal insufficiency was also considered given the coexistence of hyponatremia, hyperkalemia, hypoglycemia and hypotension. However, serum cortisol was within the reference range, abdominal CT showed normal adrenal glands, and after endocrinology consultation, further adrenal testing was not deemed necessary.

In view of symptomatic severe hyponatremia, hypoglycemia and hyperkalemia, urgent resuscitation and supportive care were initiated. Hyperkalemia was treated with 5 IU of insulin in 500 mL of dextrose solution, administered with concomitant glucose supplementation given the coexisting hypoglycemia; serum potassium decreased to 4.8 mmol/L by day 1. Serum sodium was corrected gradually with isotonic saline, keeping the correction rate below 10 mmol/L per 24 hours: 98 mmol/L on day 1, 102 on day 2, 105 on day 3, 111 on day 4, 120 on day 5 and 128 mmol/L on day 6. No neurological deterioration suggestive of osmotic demyelination and no cardiac arrhythmia occurred during correction. Despite repeated glucose supplementation, recurrent hypoglycemic episodes persisted for approximately 10 days. The patient showed progressive clinical improvement; notably, these abnormalities improved without glucocorticoid therapy.

As part of the pre-procedure workup, a coagulation panel showed an isolated prolongation of the aPTT (ratio 1.4) with a normal prothrombin time (70%) and a serum fibrinogen of 5 g/L (mildly elevated). Hematology specialists recommended fresh frozen plasma (FFP) transfusion; however, the aPTT remained prolonged after transfusion. To investigate the prolonged aPTT, a 1:1 mixing study with normal pooled plasma was performed, yielding a Rosner index of 15.4%, above the laboratory's cutoff of 15%. This result indicated a lack of correction suggestive of an inhibitor, consistent with the absence of correction after FFP transfusion, although the value was close to the threshold (Table 1). Coagulation factor assays and specific inhibitor testing were not performed.

**Table 1:** Laboratory findings

Parameter	Patient value	Reference range
Biochemistry		
Serum sodium	95 mmol/L	135–145 mmol/L
Serum potassium	6.81 mmol/L	3.5–5.0 mmol/L
Blood glucose	0.3 g/L	0.70–1.10 g/L
Plasma osmolality	201.61 mOsm/kg	275–295 mOsm/kg
24-hour urinary sodium	150 mmol/24h	40–220 mmol/24h
Plasma uric acid	0.5 mg/dL	3.5–7.2 mg/dL
Serum cortisol (AM)	358 nmol/L	140–690 nmol/L
TSH	0.53 mIU/L	0.4–4.0 mIU/L
Coagulation panel		
aPTT ratio (patient/control)	1.4	0.8–1.2
Prothrombin time	70%	70–100%
Serum fibrinogen	5 g/L	2–4 g/L
Mixing study (Rosner index)	15.4%	≤15%: correction; >15%: suggests inhibitor (laboratory cutoff)

Antinuclear antibodies, anti-dsDNA antibodies, anticardiolipin and anti-β2-glycoprotein I antibodies were negative, and lupus anticoagulant testing was negative, providing no serological evidence of the evaluated autoimmune conditions (Table 2).

**Table 2:** Serological findings and their interpretation *The specific functional assay used for lupus anticoagulant testing was not available in the patient's records.

Test	Result	Reference range	Interpretation
Antinuclear antibodies (ANA)	Negative	Negative (<1:80)	No serological evidence of connective tissue disease
Anti-dsDNA antibodies	Negative	Negative	Argues against systemic lupus erythematosus
Anticardiolipin and anti-β2-glycoprotein I antibodies	Negative	Negative	No serological support for antiphospholipid syndrome
Lupus anticoagulant*	Negative	Negative	No evidence of lupus anticoagulant

A thoracic CT scan revealed a right apical oval-shaped tissue lesion with irregular margins, measuring 41 × 30 × 38 mm, and mediastinal lymphadenopathies, the largest in the 2R station measuring 13 mm. Apical paraseptal emphysema was noted, with no pleural or pericardial effusion or suspicious thoracic bone lesions (Figures 2, 3).

**Figure 2:**
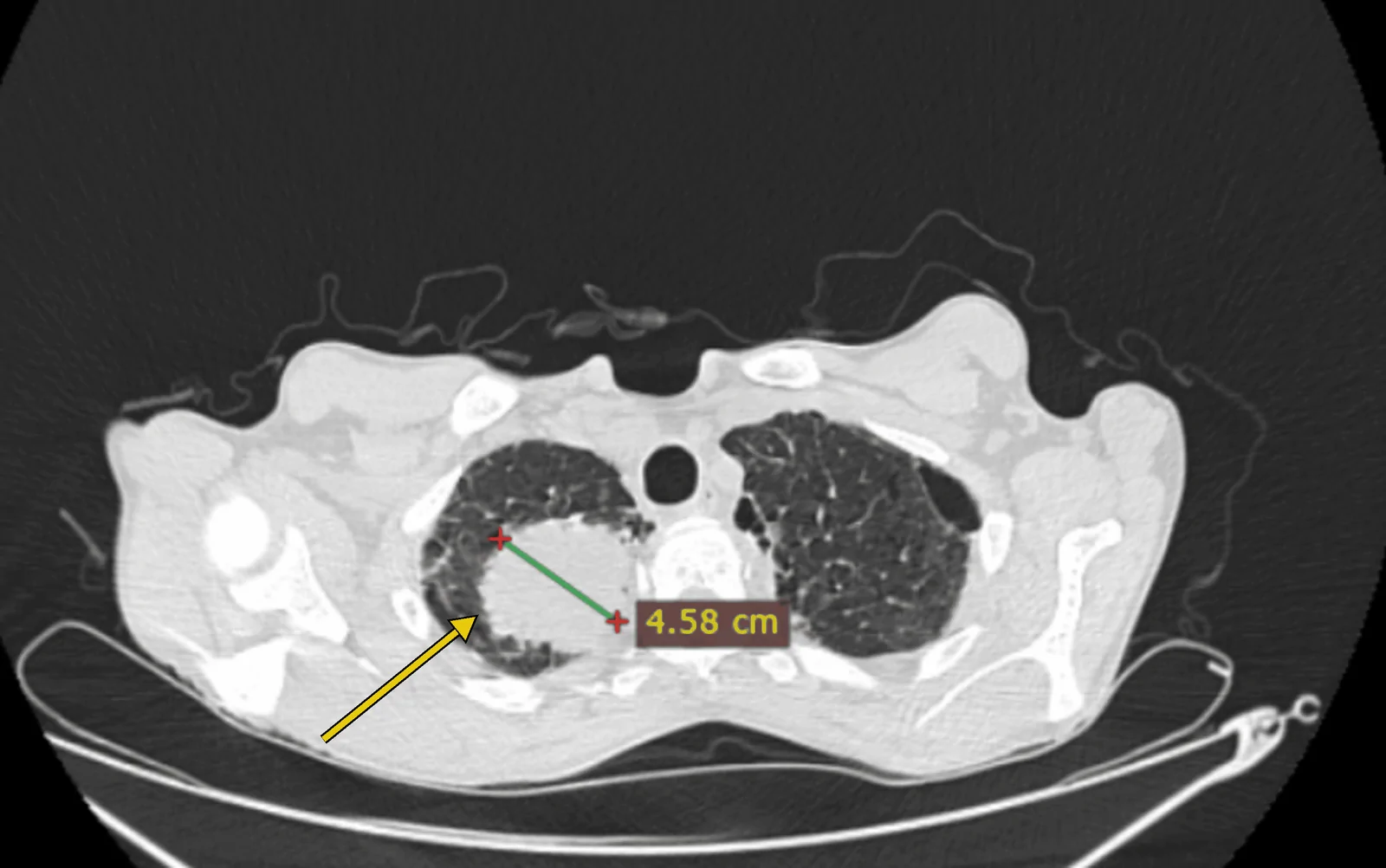
Axial contrast-enhanced chest CT (lung window) showing an oval right upper lobe mass with irregular margins (arrow). The measurement shown corresponds to the maximal oblique diameter on this slice.

**Figure 3:**
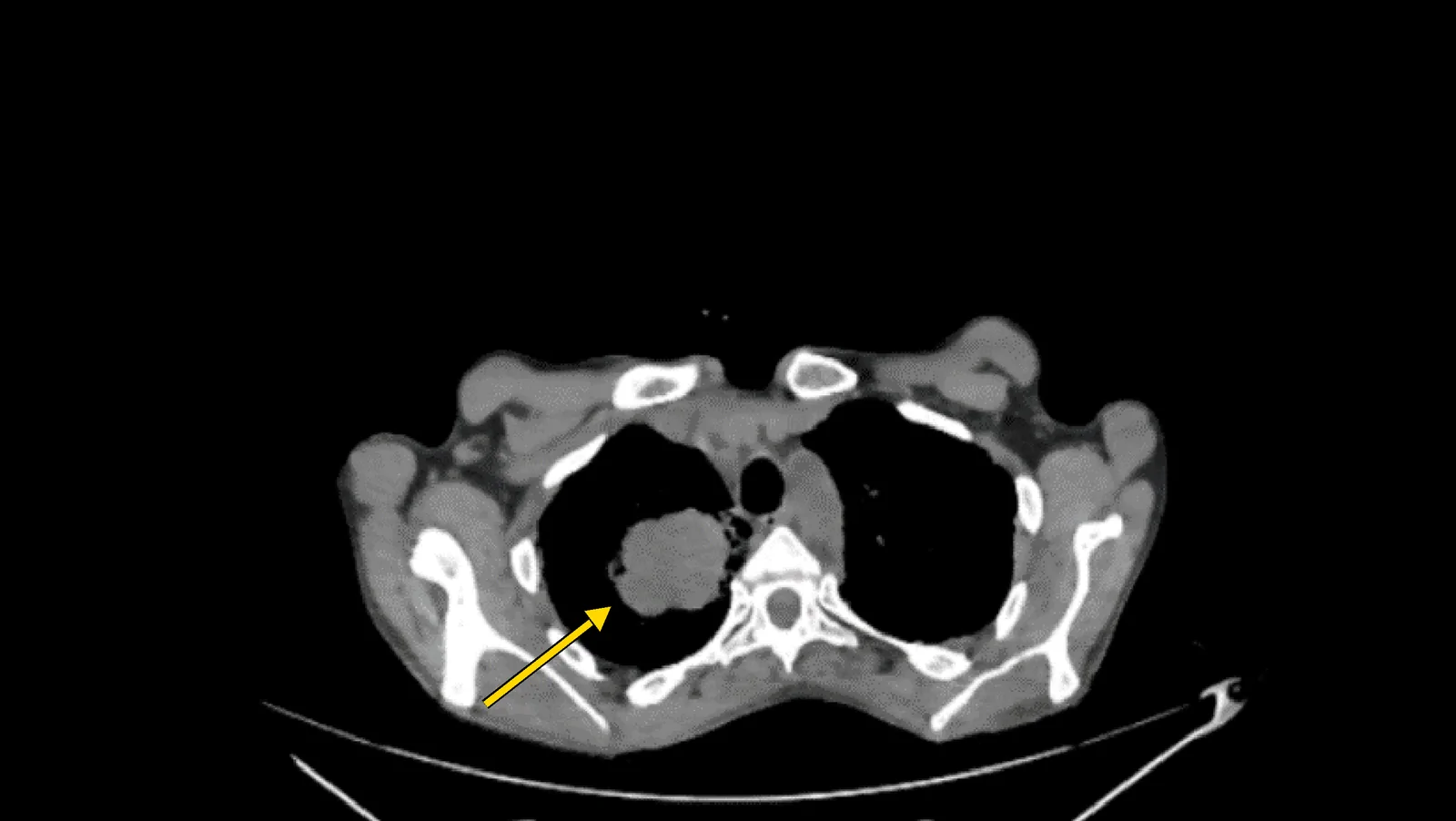
Axial contrast-enhanced chest CT (mediastinal window) demonstrating the right upper lobe mass (arrow).

In the absence of bleeding symptoms or bleeding history, and given the lack of correction after FFP transfusion, the hematology team considered the procedural bleeding risk acceptable, and CT-guided biopsy of the lesion was performed without specific precautions. No bleeding complications occurred, and the aPTT remained prolonged after the procedure. A paraneoplastic origin of the prolonged aPTT was suspected; however, it could not be definitively established without assessment of coagulation-factor activity and potential inhibitors.

Histopathological examination of the biopsy revealed a pulmonary adenocarcinoma; immunohistochemistry showed strong positivity for CK7 and aberrant cytoplasmic staining for TTF-1 (Figure 4). Staging workup, including abdominal and brain CT, showed no distant metastases. The tumor was staged as T2bN2aM0 (stage IIIA) according to the 9th edition of the TNM classification. The patient was referred to the oncology center for chemotherapy and was subsequently lost to follow-up; therefore, the evolution of the biochemical and coagulation abnormalities after cancer treatment could not be assessed.

**Figure 4:**
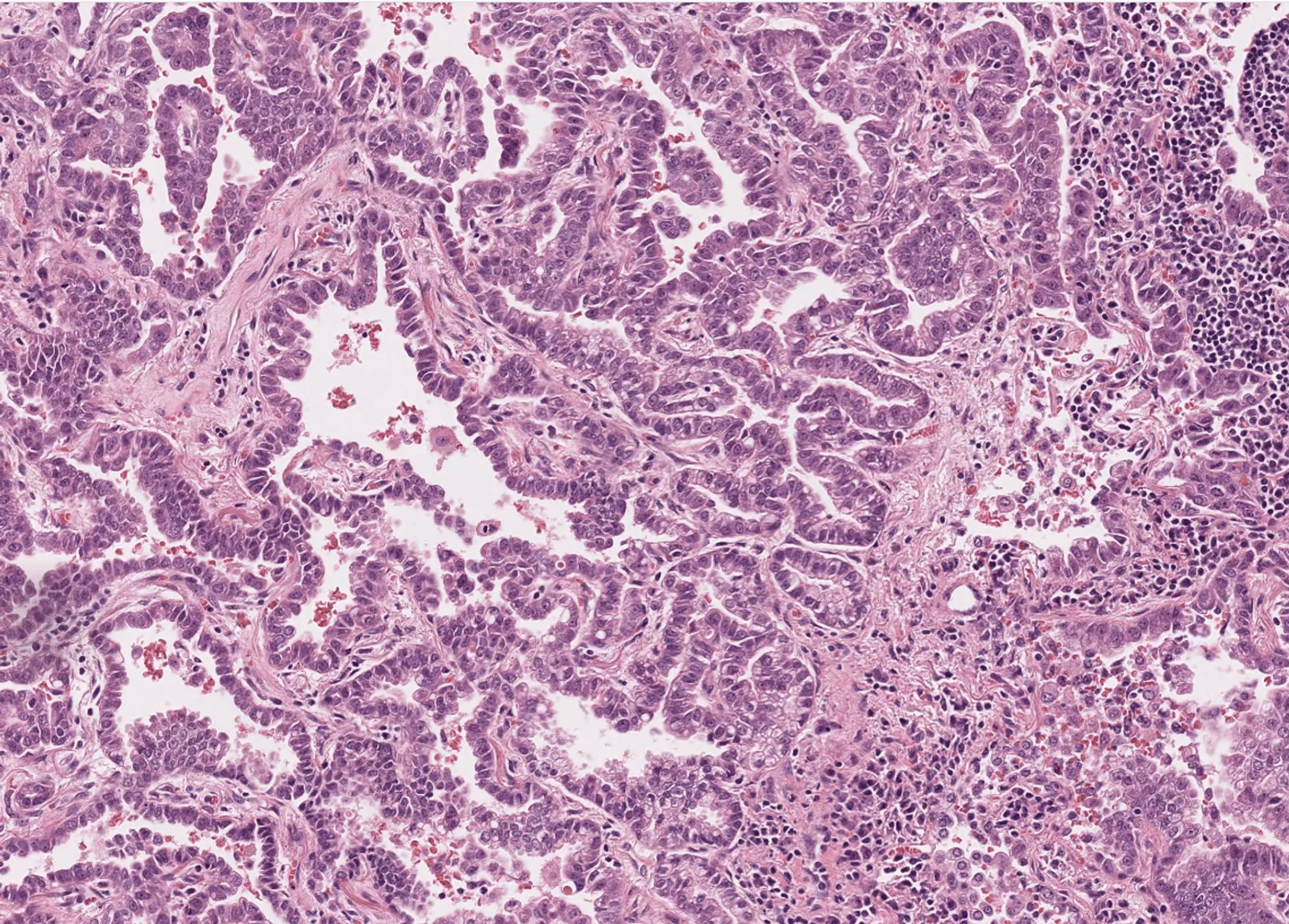
Microphotograph (hematoxylin and eosin stain) showing a carcinomatous proliferation arranged in papillary and irregular glandular structures with marked atypia, consistent with invasive lung adenocarcinoma. Immunohistochemistry showed strong CK7 positivity and aberrant cytoplasmic TTF-1 staining.

## Discussion

Lung cancer is the leading cause of cancer mortality in the world, with approximately 1.8 million deaths per year [2]. Approximately 10% to 20% of lung cancer patients experience paraneoplastic syndromes, which encompass various clinical disorders caused by substances such as hormones or antibodies secreted by tumors [12]. These syndromes occur without direct tumor growth or infiltration and are not due to distant metastases [4,5].

Severity of symptoms does not correlate with the size of the primary tumor or the stage of the disease. Although specific diagnostic criteria have been proposed (Table 3), not all are necessarily required to be met in routine clinical practice [5]. In our patient, only some of these criteria could be assessed (Table 3), which is why the paraneoplastic origin of the abnormalities remains presumptive.

**Table 3:** Diagnostic criteria for paraneoplastic endocrine syndromes and their assessment in this case. Criteria adapted from Upadhya et al. [5].

Criterion	In this case
1	Endocrine function abnormality with absent physiologic feedback regulation	Suggested (hypotonic hyponatremia with low plasma uric acid), but not confirmed (urine osmolality unavailable)
2	The respective endocrine gland should not have any metastasis	Met (no distant metastases on abdominal and brain CT)
3	Worsening not explained by increasing tumour burden	Not assessable
4	Cancer treatment improves the endocrine function	Not assessable (lost to follow-up)
5	Tumour biopsy sample shows evidence of either the hormone substance or its production	Not performed

Humoral hypercalcemia, along with SIADH, is the most common endocrine paraneoplastic syndrome [5,7]. Hypercalcemia arises primarily through two pathogenic mechanisms: osteolytic activity secondary to bone metastases, and humoral hypercalcemia mediated by tumor production of parathyroid hormone-related protein (PTHrP). The severity of clinical manifestations correlates with serum calcium levels and can include cardiovascular dysfunction, gastrointestinal disturbances, and neuromuscular and neuropsychiatric alterations [5].

Hyponatremia is common in lung cancer, and registry data suggest that up to 15% of cases are caused by euvolemic SIADH. SIADH related to a malignant tumor is often latent and can lead to water intoxication symptoms such as headaches, seizures and coma, with dilutional hyponatremia and low plasma osmolality [6]. In our patient, SIADH was suspected and supported by hypouricemia, but the diagnosis could not be confirmed in the absence of urine osmolality and spot urine sodium measurements, and hypovolemia related to gastrointestinal losses may have contributed. Cerebral salt wasting was considered unlikely in the absence of brain metastases or known intracranial disease. Finally, a single serum cortisol of 358 nmol/L measured during severe hypotension may not fully exclude relative adrenal insufficiency, although the adrenal glands were normal on CT and the abnormalities improved without glucocorticoid therapy.

A prolonged aPTT in a neoplastic condition is a rare situation that requires multiple investigations to assess the specific etiology. The mixing test combines equal amounts of patient plasma and normal pooled plasma. Normalization suggests a factor deficiency, while a lack of correction suggests the presence of an inhibitor. An inhibitor could be a lupus anticoagulant, which has the properties of an antiphospholipid antibody and interferes with phospholipid-containing coagulation tests, notably by prolonging the aPTT; it tends to cause in vivo thrombosis rather than bleeding diathesis [9,10]. In our patient, the mixing study suggested an inhibitor, but coagulation factor activity (factors VIII, IX, XI and XII) and specific inhibitor assays were not performed, and the lupus anticoagulant assay method could not be specified; therefore, other causes of isolated aPTT prolongation, including factor deficiencies and acquired inhibitors, could not be fully excluded.

Severe hypoglycemia may occur in association with malignancy through several mechanisms, including increased glucose consumption and tumor-associated production of insulin-like growth factor II (IGF-II). Non-islet-cell tumor hypoglycemia has mainly been described in three tumor types: mesenchymal abdominal or thoracic tumors (Doege-Potter syndrome), adrenocortical tumors (Anderson syndrome), and primary or secondary liver tumors (Nadler-Wolffer-Elliott syndrome), although other tumors may occasionally be involved. These tumors are almost always large, and their excessive glucose consumption contributes to hypoglycemia. The tumor is also usually responsible for producing a hypoglycemic factor, IGF-II, which shares homology with IGF-1 and with insulin and binds to the insulin receptor, hence its hypoglycemic effect [7,8]. In our patient, hypoglycemia recurred for approximately 10 days despite repeated glucose supplementation, which may suggest a tumor-related mechanism. However, insulin, C-peptide, β-hydroxybutyrate and IGF-related measurements were not performed; therefore, non-islet-cell tumor hypoglycemia could not be confirmed, and severe cachexia may also have contributed.

## Conclusions

Although lung cancer continues to be the leading cause of cancer deaths worldwide, advances in survival rates are expected to lead to an increase in paraneoplastic syndromes. These syndromes may serve as indicators for prognosis and treatment response.

This case highlights the importance of considering an underlying malignancy when multiple unexplained biochemical and coagulation abnormalities coexist. In our patient, severe hyponatremia, recurrent hypoglycemia and an isolated prolonged aPTT coincided with newly diagnosed invasive lung adenocarcinoma. A systematic diagnostic evaluation is necessary to establish the cause of each abnormality and determine whether it is paraneoplastic. Given the missing investigations and the lack of follow-up, the paraneoplastic origin of these abnormalities remains presumptive. Confirming true paraneoplastic causality would require complete endocrine and hemostatic investigations and demonstration of their resolution after tumor-directed therapy.
